# Merkel cell carcinoma on the right calf in association with chronic lymphocytic leukemia: A case report

**DOI:** 10.1002/ccr3.4498

**Published:** 2021-07-06

**Authors:** Hamideh Mohammadzadeh, Sepideh Babaniamansour, Mohammadreza Majidi, Abolfazl Zare, Mohammad Dehghani Firouzabadi, Sepideh Karkon‐Shayan

**Affiliations:** ^1^ Department of Dermatology Gonabad University of Medical Sciences Gonabad Iran; ^2^ Department of Pathology School of Medicine Islamic Azad University Tehran Faculty of Medicine Tehran Iran; ^3^ Student Research Committee Faculty of Medicine Gonabad University of Medical Sciences Gonabad Iran; ^4^ Social Development and Health Promotion Research Center Gonabad University of Medical Sciences Gonabad Iran; ^5^ Head & Neck Research Center The Five Senses Health Institute Iran University of Medical Sciences Tehran Iran

**Keywords:** chronic lymphocytic leukemia, neuroendocrine tumors, Merkel cell carcinoma, prognosis

## Abstract

This study showed a rare case of Merkel cell carcinoma (MCC) with atypical manifestations accompanied by chronic lymphocytic leukemia of B‐cell type that underwent chemotherapy and had poor prognosis. The findings suggest that the physicians should consider MCC when performing diagnosis and assess all possible associated risk factors like neoplasms to achieve good prognosis.

## Graphical abstract

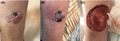

## 1 INTRODUCTION

Merkel cell carcinoma (MCC) is a rare tumor with a neuroendocrine origin. It presents as a single red nodule in the sun‐exposed area by rapid and aggressive growth. This study presented a rare case of MCC on an unusual site associated with chronic lymphocytic leukemia.

Merkel cell carcinoma is a rare tumor with an aggressive neuroendocrine origin. MCC arises from Merkel cells of the basal layer of epidermis or hair follicles, stem cells of the dermis, or precursor B cell.^1^ In the meantime, a study in 2008 declared that MCC could also have an infectious origin, and Merkel cell polyomavirus, a double‐strand DNA virus of the Polyomaviridae family, was a causative agent.^2^, ^3^ About 0.13 to 1.6 per 100,000 persons are annually diagnosed with MCC, worldwide.^4^ White elders, those under immunosuppressive treatments or who have other malignancies, are more vulnerable to MCC. MCC mostly occurs as a painless red or purple nodule or solitary plaque, in the sun‐exposed area (43% at head and neck, 24% at upper limbs and shoulders), with rapid and aggressive growth and poor prognosis.^1^, ^5^

Merkel cell carcinoma is more common in men and the seventies. It presents as local involvements (65%), regional lymph node metastasis (26%), and distant metastasis (8%) and has a 30% mortality rate worldwide. In this regard, diagnostic management of these patients requires the entire body examination of the lymph nodes and all required laboratory tests or imaging to rule out differential diagnoses such as basal cell carcinoma (BCC), squamous cell carcinoma, adnexal tumor, amelanotic melanoma, epidermoid cysts, pyogenic granuloma, angiosarcoma, and lymphoma. Consequently, it needs combined therapeutic management.^1^, ^6^ This study aimed to present a rare case of MCC, on an unusual site, associated with chronic lymphocytic leukemia (CLL) and its prognosis.

## 2 CASE PRESENTATION

### 2.1 Case history/examination

**FIGURE 1:**
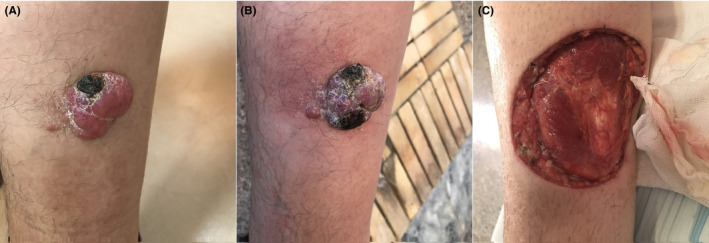
Pigmented papillomatosis nodule on the inner region of the right calf (A: first visit, B: second visit, C: after surgery)

A 69‐year‐old Caucasian man (from the northeastern part of Iran) reported to the clinic of dermatology with a chief complaint of a single lesion on the inner region of his right calf from 6 months ago. The lesion was a firm, violaceous, erythematous, and eroded nodule, which was enlarged three‐fold during last month and caused discomfort. It was 3.5 × 3.5 × 1.5 cm, sharp margin, stuck to the skin, and exhibiting a pigmented papillomatosis surface. On the other hand, the lesion was painless, non‐pruritic, non‐hemorrhagic, no secretion, or smell. No color change was reported. No tenderness was observed on touch (Figure 1).

Considering the past medical history showed a history of BCC and Seborrheic keratosis on the face. Besides, there was no history of smoking, alcohol consumption, or family history of skin disease or malignancies.

There was no evidence of significant weight loss, abnormal range of vital signs, fever, or any other signs and symptoms.

### 2.2 Differential diagnosis, investigations, and treatment

**FIGURE 2:**
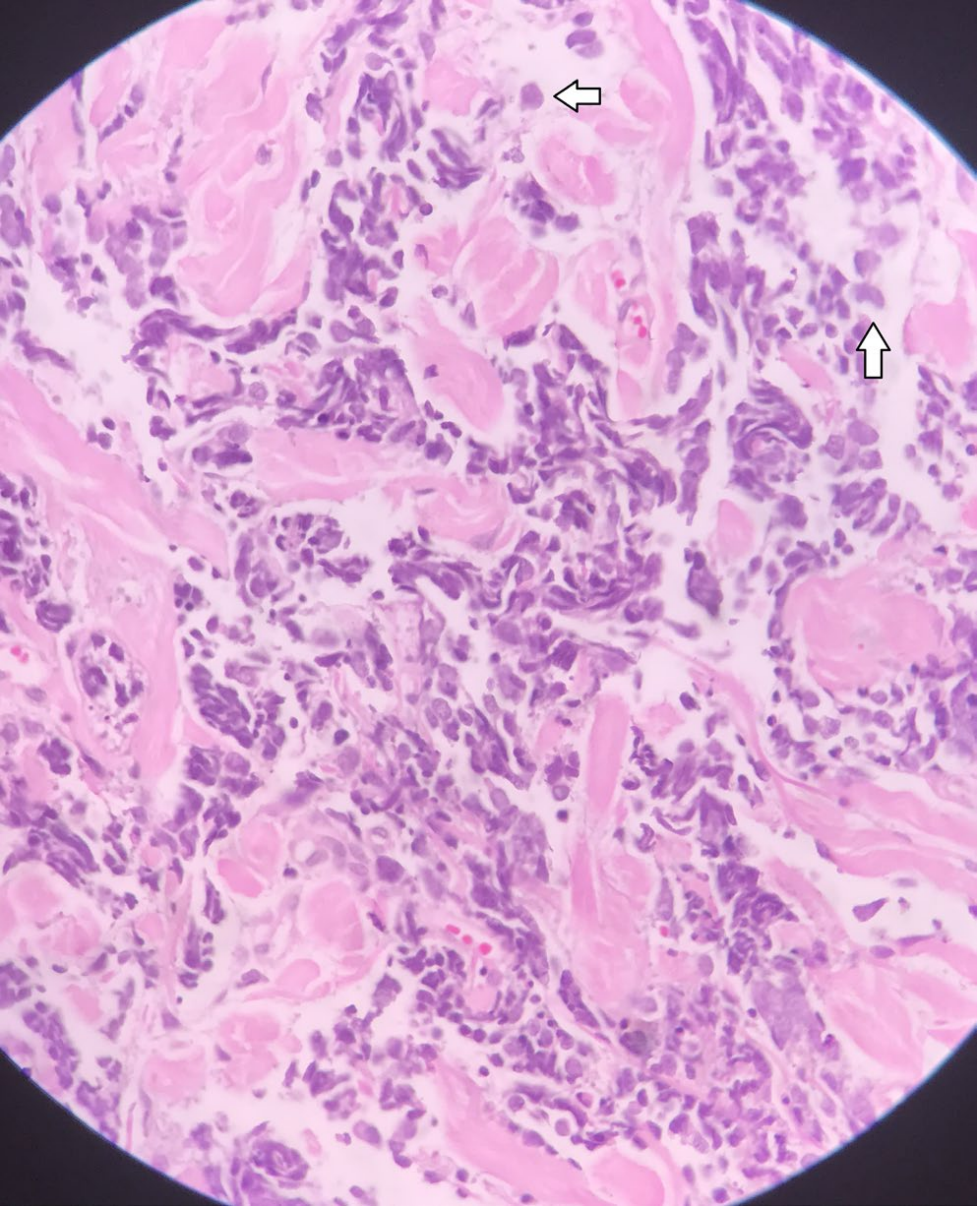
Microscopic examination revealed a small round cell tumor. Orthokeratotic epidermis, the lower layer of the dermis with sheets of cells with high nucleus‐cytoplasmic ratio (white arrows), hyperchromatic nuclei, and increased mitotic activity (HE ×400)

**FIGURE 3:**
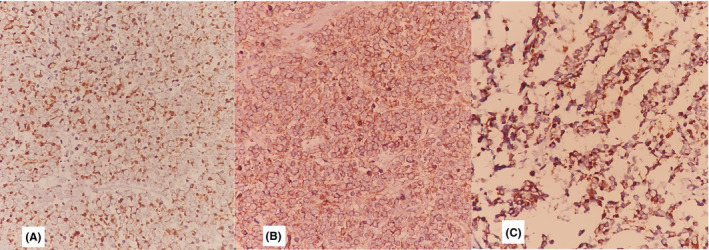
Immunochemical features showed that the neuroendocrine component was immunoreactive for (A) synaptophysin, (B) CD 56, (C) CK 20 (original magnification ×20)

In the following, a 2 × 3 mm section with a 1 cm healthy margin was excised for pathological evaluation and labeled as excisional shine lesion biopsy. It showed that the skin section was accompanied by an orthokeratotic epidermis. Also, a lower layer of the dermis with cell sheets, high nucleus‐to‐cytoplasm ratio, hyperchromatic nuclei, and briefly pleomorphic with mitotic views, penetrating the collagen bundles of hypodermis, and fat tissue were evident. A diagnosis of a small round cell tumor was given (Figure 2). Differential diagnoses of MCC, B‐cell lymphoma (BCL), melanoma, and also thyroid malignancy with skin manifestations were made with the preference of MCC and recommended immunohistochemistry (IHC) staining. A panel of IHC staining demonstrated positive stains for chromogranin, neuron‐specific enolase (NSE), cytokeratin (CK20), CD 56, and synaptophysin (Figure 3). It also reported negative stains for transcription termination factor 1 (TTF‐1) (rule out thyroid malignancies), S100 protein (rule out melanoma), and B‐lymphocyte antigen (CD 20) protein (rule out BCL).

The ultrasound of the right groin was revealed the presence of multiple lymph nodes with thick and hypervascular cortex, and central and peripheral vascular flow (the largest lymph node was 27 × 19 × 31 mm), which strongly suggestive of malignancy. In this regard, 45 lymph nodes at the right inguinal with a 20 × 10 × 5 cm area were removed by a plastic surgeon. The biopsy of lymph nodes confirmed the MCC involvements.

A spiral computed tomography (CT) scan of the abdomen with intravenous (IV) and oral contrast showed no findings in favor of pulmonary involvement. A maximum transverse diameter of 34 mm was reported around the inferior vena cava (IVC), causing the IVC to move anteriorly and narrow the stenosis. No clear lytic or bony blast lesion was reported.

Laboratory tests demonstrated WBC = 61.500 (Neutrophil = 7.4, lymphocyte = 85.5), Hb = 9.7, Hct = 31.9, MCV = 85, MCH = 25.7, MCHC = 30.3, RDW = 14.4, ALKP = 323, and LDH = 1981. The peripheral blood smear test showed the smudge cell, suggestive of lymphoproliferative disorders following CLL, and recommended immunophenotyping. Therefore, Flow cytometry reported lymphoproliferative B‐cell disorder with 88% B cell and 3% T cell. Immunophenotyping noted markers of CD 5+, CD 19+, CD 20 +, and CD 23 +, which was suggestive of CLL of B‐cell type.

### 2.3 Outcome and follow‐up

According to the American Joint Committee of Cancer (AJCC) staging system, the clinical stage of MCC lesion in this study was 4 (T2N3M1). The patient was scheduled for chemotherapy medications including IV Etoposide (120 mg/day) and IV Cisplatin (150 mg/day) for 3 days repeated every 3 weeks. Three months later, a thoracic contrast‐enhanced CT scan showed multiple adenopathies with large calcification (transverse diameter of 15 mm in the mediastinum) and ninety prominent lymph nodes on the axillary ligaments of both sides. Scattered sclerotic lesions were observed in the dorsal part of metastatic lumbar vertebrae, right iliac wing, middle sacrum, right femoral head, and inferior ramus of the left pubis. Despite all diagnostic and treatment procedures performed for this patient, he died after 9 months which may be due to the old age, gender, rapid growth and metastasis of MCC, and its association with several cancers and skin disease.

## 3 DISCUSSION

Merkel cell carcinoma is a rare skin neoplasm with a high mortality rate. It has wide different histopathologic features, high number of missed diagnosed cases, which can be associated with its uncertain origin. Besides, it has aggressive behavior and rapid growth that leads to multidisciplinary approaches. IHC and lymph node evaluation play important roles in MCC management. MCC is composed of round tumor cells with high nuclei to cytoplasm ratio. It mostly stains positive for CK epithelial membrane antigen and neuroendocrine markers such as NSE, chromogranin, synaptophysin, and CD56. Among these, CK20 is more specific for MCC.^1^, ^7^ Besides, terminal deoxynucleotidyl transferase immuno‐histo‐staining can strengthen MCC diagnosis.^8^ The recommended treatment for MCC is wide local excision with a healthy margin of 1–3 cm with adjacent therapies. Studies showed that MCC has a local recurrence, local lymph node metastasis, and distant metastasis rates of 20%–75%, 31%–80%, and 26%–75%, respectively.^9^, ^10^ Studies asserted that the recurrence rate of MCC in those with the previous history of MCC lesions was in the range of 22%–200%.^11^, ^12^ Risk factors for bad prognosis were as follows: old of age, males, primary size of MCC more than 2 cm, location in the lower extremities, lymph node involvement, and late referral of patient (more than 3 months).^13^, ^14^

On the other hand, CLL is the most common leukemia in adults with B‐lymphocyte origin with an incidence rate of three to five cases per 100,000 persons.^15^ Previous studies reported several cases of MCC and CLL, but the cause of this association between CLL and MCC is still a matter of debate. Some studies stated that Merkel cell polyomavirus, a causative agent of MCC, can be a possible link for the pathogenesis of CLL.^2^ Barroeta and Farkas^16^ reported a middle‐aged man with a history of CLL and simultaneous involvement of the arm by CLL and primary MCC. Antic et al. presented an untreated case of CLL with an MCC lesion on the right gluteal region. They declared that 6 months after the surgery of the MCC lesion, the presentation of lymphadenopathy at the same site confirmed the MCC relapse.^15^ Besides, Popovic et al.^17^ reported a non‐immunosuppress host of CLL with secondary MCC. On the other hand, a study among 1306 patients with MCC and 2048739 patients with other primary cancers showed that those with primary MCC were at 1.22 times higher risk of developing subsequent cancers (at salivatory, liver, or gallbladder), especially at the first year of diagnosis but CLL was not significantly associated with primary MCC. However, those with other malignancies (especially with CLL) were at 1.36 times higher risk of developing secondary MCC.^18^, ^19^ Koljonen et al.^20^ assessed the association between CLL and other malignancy among 4164 patients with CLL and declared that although CLL was significantly associated with various other malignancies, concomitant MCC was only observed in six patients.

This study aimed to present a rare case of MCC in a non‐sun‐exposed site, accompanied by CLL. This brings up the importance of contaminant malignancies associated with primary or secondary MCC and raises awareness about CLL‐associated neoplasms to enhance the prognosis.

## 4 CONCLUSION

Merkel cell carcinoma is a rare and mostly missed tumor with poor prognosis. In this study, we presented an MCC case with atypical presentations accompanied by CLL that had poor prognosis. The findings showed that the physicians should enhance the physicians' knowledge about MCC and its companions. Therefore, they could make the best and early diagnostic and therapeutic management even in those with atypical manifestations to achieve good prognosis.

## CONFLICT OF INTEREST

None declared.

## AUTHOR CONTRIBUTION

All authors contributed to the study's conception and design. SK‐S, AZ, SB, and HM performed material preparation, data collection, and acquisition. SB, MM, and MDF involved in writing the first draft of the manuscript. All authors read and approved the final manuscript.

## ETHICAL APPROVAL

This study was approved by the Ethics Committee of Gonabad University of Medical Sciences, Gonabad, Iran. Additionally, informed consent was obtained from the patient.

## Data Availability

The data that support the findings of this study are available from the corresponding author upon reasonable request.
